# Supplementary material for: Three new species of Acalypha L. (Euphorbiaceae, Acalyphoideae) from Tanzania and Angola and their conservation status
Source: PLoS One. 2025 Oct 29;20(10):e0332588. doi: 10.1371/journal.pone.0332588 (PMC12571263; doi:10.1371/journal.pone.0332588)
Supplement: S1 Table — (DOCX) [file pone.0332588.s002.docx]

Supplementary file 1. Extraction protocol using the CTAB method, adapted from Doyle (1991)

| **Steps** |
| --- |
| **1.** CTAB buffer per sample: 0.02 ml CTAB (2% p/v); 0.02 ml PVP [Pm 40,000] (2% p/v); 0.28 ml NaCl (1.4 M); 0.04 ml EDTA pH 8 (20 mM); 0.1 ml Tris-HCL pH 8 (100 mM); 0.02 ml 2-mercaptoetanol (2% v/v); 0.56 ml H_2_O  Add the solid components over the liquids and heat at 65 °C for 1 h |
| **2.** Grind 15 mg of dehydrated tissue in TissueLyser II (Quiagen) for 10 min |
| **3.** Add 950 μl of CTAB buffer to each tube containing the ground sample and mix thoroughly |
| **4.** Incubate the tubes in a thermoblock at 65°C for 1 hour with constant agitation at 650-700 rpm |
| **5.** Add 700 μl of chloroform (isoamyl-alcohol 24:1) to each tube |
| **6.** Centrifuge at 13,000 rpm for 15 min at 4°C |
| **7.** Transfer the supernatant to a new tube |
| **8.** Add 550 μl of isopropanol (stored in the freezer) to each tube and homogenize |
| **9.** Store at -20°C for 24-48 h |
| **10.** Centrifuge the tubes for 15 min at 13,000 rpm at 4°C |
| **11.** Carefully discard the supernatant, ensuring the pellet remains intact |
| **12.** Add 500 μl of 70% ethanol to each tube and vortex until the pellet is fully resuspended |
| **13.** Repeat step 10 |
| **14.** Repeat step 11 |
| **15.** Repeat step 12 |
| **16.** Repeat step 10 |
| **17.** Discard the liquid and incubate the open tubes at 37°C for 1 hour until completely dry |
| **18.** Resuspend the pellet in 50 μl of preheated (65°C) MilliQ water and store at 4°C |
