# Supplementary material for: Three new species of Acalypha L. (Euphorbiaceae, Acalyphoideae) from Tanzania and Angola and their conservation status
Source: PLoS One. 2025 Oct 29;20(10):e0332588. doi: 10.1371/journal.pone.0332588 (PMC12571263; doi:10.1371/journal.pone.0332588)
Supplement: Attachment — Submitted filename: PONE-D-25-22700_REV.pdf [file pone.0332588.s004.pdf]

PLOS ONE

Three new species of Acalypha L. (Euphorbiaceae, Acalyphoideae) from Tanzania and Angola and their conservation status

--Manuscript Draft--

**Manuscript Number:**	PONE-D-25-22700
**Article Type:**	Research Article
**Full Title:**	Three new species of Acalypha L. (Euphorbiaceae, Acalyphoideae) from Tanzania and Angola and their conservation status
**Short Title:**	New species of Acalypha (Euphorbiaceae) from Tanzania and Angola and their conservation status
**Corresponding Author:**	Pablo Muñoz Rodríguez<br>Universidad Complutense de Madrid<br>Madrid, Madrid SPAIN
**Keywords:**	Africa; conservation; herbarium collections; species discovery; taxonomy
**Abstract:**	We describe <i>Acalypha brevipetiolata</i> and <i>A. bracteolata</i> from Tanzania, and <i>A. linearis</i> from Angola, as species new to science. We provide illustrations, distribution maps, and preliminary conservation assessments. We discuss their significance in the context of both countries and within the framework of an ongoing taxonomic monograph of the genus in Africa. These are the first new <i>Acalypha</i> species described in Africa in nearly two decades, in Tanzania in five decades, and in Angola in over a century.
**Order of Authors:**	Iris Montero-Muñoz
	José María Cardiel
	Lucía Villaescusa-González
	Pablo Muñoz Rodríguez
**Additional Information:**	
**Question**	**Response**
**Financial Disclosure**<br><br>Enter a financial disclosure statement that describes the sources of funding for the work included in this submission. Review the submission guidelines for detailed requirements. View published research articles from *PLOS ONE* for specific examples.<br><br>This statement is required for submission and **will appear in the published article** if the submission is accepted. Please make sure it is accurate.	Yes

Powered by Editorial Manager® and ProduXion Manager® from Aries Systems Corporation

**Funded studies**<br>Enter a statement with the following details:<br>• Initials of the authors who received each award<br>• Grant numbers awarded to each author<br>• The full name of each funder<br>• URL of each funder website<br>• Did the sponsors or funders play any role in the study design, data collection and analysis, decision to publish, or preparation of the manuscript?<br><br>Did you receive funding for this work?	
Please add funding details.<br>as follow-up to "**Financial Disclosure**<br><br>Enter a financial disclosure statement that describes the sources of funding for the work included in this submission. Review the submission guidelines for detailed requirements. View published research articles from *PLOS ONE* for specific examples.<br><br>This statement is required for submission and **will appear in the published article** if the submission is accepted. Please make sure it is accurate.<br><br>**Funded studies**<br>Enter a statement with the following details:<br>• Initials of the authors who received each award<br>• Grant numbers awarded to each author<br>• The full name of each funder<br>• URL of each funder website<br>• Did the sponsors or funders play any role in the study design, data collection and analysis, decision to publish, or preparation of the manuscript?<br><br>Did you receive funding for this work?"	The authors are grateful for the financial support provided by the research project PID2022-139634NA-I00M funded by MCIN/AEI/10.13039/501100011033/FEDER, UE. PMR was supported by a Ramón y Cajal research contract, grant ref. RYC2021-032489-I, and IMM by a Juan de la Cierva – Formación-2021, grant ref. FJC2021-046607-I, funded by MCIN/AEI/ 10.13039/501100011033 and the European Union NextGenerationEU/PRTR during the work on this project.
Please select the country of your main research funder (please select carefully as in some cases this is used in fee calculation).	SPAIN - ES

as follow-up to "**Financial Disclosure**<br><br>Enter a financial disclosure statement that describes the sources of funding for the work included in this submission. Review the submission guidelines for detailed requirements. View published research articles from *PLOS ONE* for specific examples.<br><br>This statement is required for submission and **will appear in the published article** if the submission is accepted. Please make sure it is accurate.<br><br>**Funded studies**<br>Enter a statement with the following details:<br>• Initials of the authors who received each award<br>• Grant numbers awarded to each author<br>• The full name of each funder<br>• URL of each funder website<br>• Did the sponsors or funders play any role in the study design, data collection and analysis, decision to publish, or preparation of the manuscript?<br><br>Did you receive funding for this work?"	
**Competing Interests**<br><br>Use the instructions below to enter a competing interest statement for this submission. On behalf of all authors, disclose any competing interests that could be perceived to bias this work—acknowledging all financial support and any other relevant financial or non-financial competing interests.<br><br>This statement is required for submission and **will appear in the published article** if the submission is accepted. Please make sure it is accurate and that any funding sources listed in your Funding Information later in the submission form are also declared in your Financial Disclosure statement.	The authors have declared that no competing interests exist.

Powered by Editorial Manager® and ProduXion Manager® from Aries Systems Corporation

View published research articles from *PLOS ONE* for specific examples.<br><br>**NO authors have competing interests**<br><br>Enter: *The authors have declared that no competing interests exist.*<br><br>**Authors with competing interests**<br><br>Enter competing interest details beginning with this statement:<br><br>*I have read the journal's policy and the authors of this manuscript have the following competing interests: [insert competing interests here]*<br><br>* typeset	
**Ethics Statement**<br><br>Enter an ethics statement for this submission. This statement is required if the study involved:<br><br>• Human participants<br>• Human specimens or tissue<br>• Vertebrate animals or cephalopods<br>• Vertebrate embryos or tissues<br>• Field research<br><br>Write "N/A" if the submission does not require an ethics statement.<br><br>General guidance is provided below. Consult the submission guidelines for detailed instructions. **Make sure that all information entered here is included in the Methods section of the manuscript.**	N/A

Powered by Editorial Manager® and ProduXion Manager® from Aries Systems Corporation

**Format for specific study types**<br><br>**Human Subject Research (involving human participants and/or tissue)**<br>• Give the name of the institutional review board or ethics committee that approved the study<br>• Include the approval number and/or a statement indicating approval of this research<br>• Indicate the form of consent obtained (written/oral) or the reason that consent was not obtained (e.g. the data were analyzed anonymously)<br><br>**Animal Research (involving vertebrate animals, embryos or tissues)**<br>• Provide the name of the Institutional Animal Care and Use Committee (IACUC) or other relevant ethics board that reviewed the study protocol, and indicate whether they approved this research or granted a formal waiver of ethical approval<br>• Include an approval number if one was obtained<br>• If the study involved *non-human primates*, add *additional details* about animal welfare and steps taken to ameliorate suffering<br>• If anesthesia, euthanasia, or any kind of animal sacrifice is part of the study, include briefly which substances and/or methods were applied<br><br>**Field Research**<br><br>Include the following details if this study involves the collection of plant, animal, or other materials from a natural setting:<br>• Field permit number<br>• Name of the institution or relevant body that granted permission	
**Data Availability**<br><br>Authors are required to make all data underlying the findings described fully available, without restriction, and from the time of publication. PLOS allows rare exceptions to address legal and ethical concerns. See the PLOS Data Policy and FAQ for detailed information.	Yes - all data are fully available without restriction

Powered by Editorial Manager® and ProduXion Manager® from Aries Systems Corporation

A Data Availability Statement describing where the data can be found is required at submission. Your answers to this question constitute the Data Availability Statement and **will be published in the article**, if accepted.<br><br>**Important:** Stating 'data available on request from the author' is not sufficient. If your data are only available upon request, select 'No' for the first question and explain your exceptional situation in the text box.<br><br>Do the authors confirm that all data underlying the findings described in their manuscript are fully available without restriction?	
**Describe where the data may be found in full sentences. If you are copying our sample text, replace any instances of XXX with the appropriate details.**<br><br>• If the data are **held or will be held in a public repository**, include URLs, accession numbers or DOIs. If this information will only be available after acceptance, indicate this by ticking the box below. For example: *All XXX files are available from the XXX database (accession number(s) XXX, XXX.*).<br>• If the data are all contained **within the manuscript and/or Supporting Information files**, enter the following: *All relevant data are within the manuscript and its Supporting Information files.*<br>• If neither of these applies but you are able to provide **details of access elsewhere**, with or without limitations, please do so. For example:<br><br>*Data cannot be shared publicly because of [XXX]. Data are available from the XXX Institutional Data Access / Ethics Committee (contact via XXX) for researchers who meet the criteria for access to confidential data.*<br><br>*The data underlying the results presented in the study are available from (include the name of the third party*	All relevant data are within the manuscript and its Supporting Information files. DNA sequence data has been already submitted to GenBank and assigned ID numbers, and will be released upon publication.

and contact information or URL).<br>• This text is appropriate if the data are owned by a third party and authors do not have permission to share the data.<br><br>* typeset	
Additional data availability information:	

Powered by Editorial Manager® and ProduXion Manager® from Aries Systems Corporation

Manuscript Click here to access/download;Manuscript;manuscript.docx

Three new species of *Acalypha* L. (Euphorbiaceae, Acalyphoideae) from Tanzania and Angola and their conservation status

Iris Montero-Muñoz[1], José María Cardiel[2,3], Lucía Villaescusa-González[4] & Pablo Muñoz-Rodríguez[4]*

1. Real Jardín Botánico, CSIC. Plaza de Murillo, 2. Madrid, SPAIN

2. Universidad Autónoma de Madrid, Faculty of Sciences, Department of Biology, C/ Darwin, 2. 28049 Madrid, SPAIN.

3. Centro de Investigación en Biodiversidad y Cambio Global (CIBC-UAM), Universidad Autónoma de Madrid. C/ Darwin, 2. 28049 Madrid, SPAIN.

4. Universidad Complutense de Madrid, Faculty of Biological Sciences, Department of Biodiversity, Ecology and Evolution. C/ José Antonio Novais, 12. 28040 Madrid, SPAIN.

*Corresponding authors: pablo.munoz@ucm.es

ABSTRACT

We describe *Acalypha brevipetiolata* and *A. bracteolata* from Tanzania, and *A. linearis* from Angola, as species new to science. We provide illustrations, distribution maps, and preliminary conservation assessments. We discuss their significance in the context of both countries and within the framework of an ongoing taxonomic monograph of the genus in Africa. These are the first new *Acalypha* species described in Africa in nearly two decades, in Tanzania in five decades, and in Angola in over a century.

Key words

Africa, conservation, herbarium collections, species discovery, taxonomy.

1. INTRODUCTION

Acalypha L. (Euphorbiaceae Juss.) is a large plant genus with over 470 accepted species and a mainly pantropical distribution. As with many other tropical plant groups, knowledge of the genus remains uneven. *Acalypha* has been extensively studied in South America, where the authors of this paper have worked for the past three decades (Cardiel, 1995; Cardiel et al., 2023), and significant taxonomic revisions have also been conducted in the Western Indian OceanrRegion, including Madagascar (Montero-Muñoz et al., 2023), and in Southeast Asia (Sagun et al., 2010). In contrast, Africa remains one of the least well-documented regions for the genus.

Few studies have focused on *Acalypha* in Tanzania or Angola, the two countries where the new species reported here were discovered. In Tanzania, the most comprehensive floristic treatment remains Radcliffe-Smith's work for the *Flora of Tropical East Africa* (Radcliffe-Smith, 1987). The only other taxonomic work we are aware of is R.L.M. Mahunnah's PhD thesis, which questioned species boundaries within *Acalypha* but did not propose taxonomic rearrangements (Mahunnah, 1995).

The situation in Angola is even more precarious: the only available reference about *Acalypha* is a checklist lacking taxonomic information (Figueiredo and Smith, 2008). Online herbarium records for Angolan *Acalypha* are extremely limited (e.g., fewer than 300 records in GBIF), particularly for the region where our newly described Angolan species was collected, making broad taxonomic comparisons difficult. Although Tanzania has more digitised records, coverage remains sparse compared to other African countries.

In a recently published nomenclatural review (Cardiel et al., 2025) we recognised 70 native species in mainland Africa, with 28 species recorded from Tanzania and 23 from Angola. Most species have not been critically examined or had their boundaries tested since their

original descriptions —often over a century ago—, and a significant proportion (ca. 20%) are known only from type specimens or a handful of collections. The scarcity of data hampers any study on this megadiverse group of plants, emphasising the need for urgent taxonomic revision.

To address these gaps, in 2023 we started work on a continental-scale taxonomic monograph of *Acalypha* in mainland Africa. Here, we formally describe three species new to science discovered during our study of herbarium material, integrating morphological and, where possible, molecular data. We also discuss their phylogenetic and biogeographical significance in the context of *Acalypha* diversity in Africa.

2. MATERIALS AND METHODS

2.1. Taxonomic approach

We based our taxonomic decisions primarily on morphological evidence, complemented by geographical and ecological data. For one species (*Acalypha brevipetiolata*), we also obtained high-quality DNA for sequencing using the Sanger method (see below). We derived morphological descriptions and illustrations from herbarium specimens housed in BR, K, L, LISC, LISU, MA, MO, and P (abbreviations according to Thiers, 2025). We examined all cited specimens and provide herbarium identifiers when available.

To conduct comparative morphological analyses, we examined herbarium specimens of other African *Acalypha* species. We extracted information on habit, plant size, and habitat from field notes recorded on specimen labels. We followed a heuristic approach to delimit the new species (Wells et al., 2021), which allows us to recognise species boundaries based on consistent but not necessarily absolute discontinuities, ensuring taxonomic progress while accommodating potential future refinements. It also avoids rigid criteria that may lead to

over-splitting or over-lumping, making it particularly useful for taxa with limited molecular data, as is the case here.

We maintain all taxonomic and biogeographical data on *Acalypha* in the regularly updated *Acalypha Taxonomic Information System*, available at https://acalypha.es.

2.2. DNA extraction, sequencing and phylogenetic analysis

We attempted DNA extraction and amplification from multiple collections of the new species to place them in a phylogenetic context. However, despite several attempts using different methods, we obtained high-quality DNA sequences for only one specimen of *A. brevipetiolata* (*S. Bigdoog 8090*). Future efforts, possibly using fresher material, may improve sequencing success for the other taxa. Following previous studies (Levin et al., 2022; Sagun et al., 2010) and our ongoing work on *Acalypha* (unpublished), we targeted the nuclear Internal Transcribed Spacer (*nrITS*) DNA region.

We extracted DNA from herbarium specimens using the CTAB method (Doyle and Doyle, 1990) with modifications (Supplementary File 1) and quantified it using Qubit™ dsDNA BR Standard (Invitrogen). Due to DNA degradation, we amplified the *nrITS* region in two parts using primers *ITSw1f-ITSp2r* (5'-CCTTATCATTTAGAGGAAGGAG; 5'-GCCRAGATATCCGTTGCCGAG) (Silva et al., 2020) and *ITSp3f-ITSw2r* (5'-YGACTCTCGGCAACGGATA; 5'-TATGCTTAAAYTCAGCGGGT) (Cheng et al., 2016). We performed PCR reactions in a 25 µl volume, containing 12 µL of MyTaq Red Mix (Bioline), 10 µL H_2O, 1 µL of each primer (10µM), and 1 µL of genomic DNA, and standard PCR conditions: initial denaturation at 95°C for 5 minutes, followed by 36 cycles of 95ºC for 1 minute, 50°C for 1.5 minutes, and 72ºC for 45 seconds, with a final extension at 72°C for 10 minutes. We purified the PCR products using ExoSap and sent them for sequencing at

MACROGEN (Macrogen, Madrid, Spain) with the same amplification primers. We deposited the newly generated sequence in GenBank under accession number PV368855.

To infer phylogenetic relationships, we included 144 *nrITS* sequences previously generated by (Levin et al., 2022) and available on GenBank to represent *Acalypha* diversity, using *Bernardia* Houst. ex Mill., *Erythrococca* Benth., *Mareya* Baill. and *Micrococca* Benth. (one species each) as outgroups.

We first used NCBI BLAST with default settings to confirm sequence quality. We aligned the DNA sequences using MAFFT v.7.310 (*--auto --adjustdirection*) (Katoh and Standley, 2013) and refined the alignment with trimAL (*--automated1*) (Capella-Gutierrez et al., 2009). We inferred a Maximum Likelihood phylogeny with IQ-TREE 2.4.0 (random seed number 524112, one thread) (Nguyen et al., 2015), automatic model selection with ModelFinder (SYM+I+G4 based on the Bayesian Information Criterion) (Kalyaanamoorthy et al., 2017) and 1,000 ultrafast bootstrap replicates. In the resulting phylogenies, we collapsed all nodes with bootstrap support below 60% into polytomies.

2.3. Distribution maps and conservation assessments

To visualise the geographical distribution of the new species, we generated distribution maps using QGIS Desktop 3.28.4. We conducted conservation assessments following the IUCN Red List Categories and Criteria (IUCN, 2022). We calculated the Area of Occupancy (AOO) and Extent of Occurrence (EOO) using GeoCAT (Bachman et al., 2011), applying the standard 2 × 2 km grid cell size recommended by IUCN. We have not yet completed a formal IUCN assessment.

3. RESULTS AND DISCUSSION

3. 1. New species of *Acalypha*

3.1.1. Acalypha brevipetiolata

Acalypha brevipetiolata I.Montero, Cardiel & P.Muñoz, *sp. nov*. Type: Tanzania, T4, Mpanda District: 3 km S of Uzondo Camp, 1600 m, 5º31'S 30º32'E, 11 Mar 2009. *S. Bidgood, G. Leliyo & K. Vollesen 809*0 (MO [MO-3888236], holo.; BR [BR0000016110199], K, WAG [WAG.1196761], P [P00887479], iso.). Fig. 1.

Diagnosis. *Acalypha brevipetiolata* is primarily characterised by its reddish branches, very short petioles (1−3 mm), linear to linear-lanceolate leaf blades with entire to sparsely denticulate margins, and female inflorescences with a solitary, shortly pedunculated bract with an irregularly dentate margin (8–9 teeth) and with both simple and glandular trichomes.

Description. Perennial herb up to 25 cm tall, monoecious, with numerous erect to decumbent stems from a large woody rootstock. Branches reddish, pubescent with short, appressed trichomes, glabrescent when mature. Axillary buds inconspicuous. Stipules inconspicuous, promptly deciduous, up to 0.5 mm long, triangular, acuminate, sparsely hairy with simple, minute trichomes. Petioles 1−3 mm long, more or less flattened at base, subglabrous with some short, simple trichomes. Leaf blades (1.5−)2.5−4(−4.5) × (0.15−)0.2−0.4 cm, linear to linear-lanceolate, chartaceous; base acute to slightly decurrent; apex acute, and sometimes mucronate or slightly callose; margins entire to sparsely denticulate; upper surface glabrous; lower surface subglabrous with some appressed, simple trichomes on veins; venation pinnate, secondary veins 8–10 per side, prominent on lower surface. Inflorescences unisexual, axillary, in different stems. Male inflorescences up to 2.6 cm long; peduncle up to 5 mm long, pubescent with short, simple trichomes; flowers sparsely glomerate; bracts minute up to 0.8 mm long, ovate-lanceolate, glabrous. Female inflorescences with a solitary bract, shortly pedunculate, peduncle up to 0.7 mm long, bracts enlarging in fruit to c. 5 × 5 mm, pubescent with short, simple, appressed trichomes and some glandular trichomes at margin; margin

irregularly dentate, teeth 8–9, triangular-lanceolate, acute, ½–⅓ of the total bract length, two of them smaller basal teeth; bracteoles absent. Male flowers subsessile, pedicel up to 0.3 mm long, glabrous; flower buds not seen (fallen). Female flowers one per bract, sessile; sepals 3, up to 0.7 mm long, distinct, triangular-lanceolate, sparsely hairy, with short, simple trichomes; ovary c. 1 mm diameter, 3-lobed, densely pubescent with short, simple trichomes; styles 3, up to 3 mm long, distinct at base, with conspicuous central axis, each divided into 8–10 long, slender branches, sparsely hairy with some short, simple trichomes. Capsules up to 3 mm diameter, surface smooth, densely pubescent with short, simple trichomes. Seeds c. 2.1 mm diameter, globose, smooth.

Evolutionary relationships. *Acalypha brevipetiolata* belongs to a highly supported clade including *A. cupricola* Robyns ex G.A.Levin, *A. manniana* Müll.Arg., *A. polymorpha* Müll.Arg., and *A. welwitschiana* Müll.Arg. (Fig. 2 and Supplementary Fig. 1).

Distribution and habitat. *Acalypha brevipetiolata* appears to be endemic to Tanzania, specifically to the Uzondo Plateau in Mpanda District, where it was collected at an elevation of 1,600 m (Fig. 3). It inhabits seasonally inundated *Loudetia* Hochst. ex Steud. grasslands, growing on shallow sandy-peaty soil over sandstone rocks.

Etymology. The epithet refers to the length of the petiole which does not exceed 3 mm.

Conservation notes. *Acalypha brevipetiolata* is currently known from a single collection, and its population status remains unknown. We estimate its AOO at 4 km^2, while its EOO cannot be calculated due to the lack of data. The Uzondo Plateau remains largely undisturbed, but it is traversed by a recently-built road between Uvinza and Mpanda towns (Crawford et al., 2016). Plans to widen this road could increase habitat fragmentation and impact the species' habitat. Additionally, fires and logging represent potential long-term risks to the grassland ecosystem. Based on the available data, we preliminarily assess *A. brevipetiolata* as

Vulnerable (VU) under IUCN criterion D2. However, if habitat degradation due to infrastructure development, fire, or logging accelerates, the species could qualify for reassessment as Endangered (EN) or even Critically Endangered (CR) in the future.

3.1.2. Acalypha bracteolata

Acalypha bracteolata I.Montero, Cardiel & P.Muñoz, *sp. nov*. Type: Tanzania, T6, Kilombero District: Udzungwa Mountains National Park, Mang'ula Village, 680 m, 7º44’38’’S 36º53’14’’E, 31 May 1999. *G. Massawe 282* (MO[MO-3625495], holo.; MA, iso.). Fig. 4.

Diagnosis. *Acalypha bracteolata* is primarily characterised by its herbaceous habit, petioles up to 8 cm long, broadly ovate-lanceolate to elliptic-lanceolate leaf blades with pocket-shaped domatia at the base of the main veins, and terminal female inflorescences with bracts bearing simple and long glandular trichomes. The bract margins are irregularly dentate with 18–20 teeth, including a very prominent central tooth, and bracteoles up to 1.8 mm long.

Description. Perennial herb up to 2 m tall, monoecious. Branches pubescent with short, antrorsely curved, simple trichomes, glabrescent. Axillary buds perulate, perules 2, valvate, c. 0.8 mm long, triangular, subglabrous. Stipules conspicuous, up to 3.8 mm long, linear-lanceolate, with some simple trichomes up to 0.5 mm long and minute, sparse, glandular trichomes at margin. Petioles 3.2–7.5(–8) cm long, indumentum similar to that on young branches, glabrescent. Leaf blades (5.5–)6–11(–13) × 4–8(–8.5) cm, broadly ovate-lanceolate to elliptic-lanceolate, membranous; base rounded to subcordate; apex caudate, with a long acumen 1–2.3 cm long, mucronate; margins conspicuously serrate, teeth irregular, acute, usually with a minute, glandular trichome at apex; upper surface sparsely hairy with erect, simple trichomes up to 1 mm long; lower surface subglabrous, with appressed, simple

trichomes mainly on veins; venation actinodromous, basal veins 3–5(–7), secondary veins 5–7 per side, with pocket-shaped domatia at the base on the main veins.

Inflorescences unisexual, spiciform, male axillary, female terminal. Male inflorescences up to 14 cm long, densely flowered; peduncle to 1.3 cm long; flowers glomerate; bracts to 2 mm long, oblong, obtuse at apex, slightly hyaline at margin, ciliate with long, simple trichomes c. 1 mm long and some sparse, minute, glandular trichomes. Female inflorescences more or less densely flowered (with 40–50 bracts) up to 9.5 cm long; peduncle to 0.5 cm long, indumentum similar to that on young branches; bracts sessile, enlarging in fruit up to 6.2 × 4 mm, sparsely hairy with simple, short trichomes up to 0.5 mm long and long, glandular trichomes up to 0.7 mm long, mainly at margin and upper surface; margin irregularly dentate, teeth 18–20, triangular-lanceolate, acute, up to 1.5 mm long, with a very prominent central tooth up to 2.5 mm long; bracteoles up to 1.8 mm long, narrowly triangular to linear-lanceolate, with minute sessile glands at margin. Male flower with pedicel up to 0.8 mm long, glabrous; buds c. 0.6 mm diameter, glabrous. Female flower one per bract, sessile; sepals 3, up to 1.5 mm long, slightly connate at base, ovate-lanceolate, ciliate, with simple trichomes c. 0.3 mm long and some sparse sessile, glandular trichomes; ovary c. 0.7 mm diameter, 3-lobed, densely pubescent with simple trichomes up to 0.3 mm long; styles 3, up to 3.5 mm long, distinct, each divided into 8–10 slender branches, with some simple trichomes up to 0.4 mm long at rachis. Capsules present but immature. Seeds unknown.

Distribution and habitat. *Acalypha bracteolata* appears to be endemic to Tanzania, specifically to Kilombero District within Udzungwa Mountains National Park, where it was collected at an altitude of 680 m (Fig. 3). It inhabits lowland rainforest and miombo woodland.

Conservation notes. *Acalypha bracteolata* is currently known from a single collection in Udzungwa Mountains National Park, and its population status remains unknown. We estimate its AOO at 4 km², while its EOO cannot be calculated due to the limited distribution data. Udzungwa Mountains National Park has been a protected area since 1992 and is recognised for its exceptional biodiversity and high levels of endemism (Burgess et al., 2007). However, the region has experienced extensive environmental degradation, with approximately 76% of its primary vegetation lost due to logging and grazing over the past decades. While these pressures have declined since the 1970s, the spread of invasive species now poses the primary ecological threat (Doggart and Meshack, 2023).

Based on its highly restricted distribution and potential vulnerability to ongoing habitat changes, we preliminarily assess *A. bracteolata* as Vulnerable (VU) under IUCN criterion D2. However, if invasive species continue to degrade its habitat, the species could face further reassessment as Endangered (EN) or even Critically Endangered (CR) in the future.

Etymology. The epithet refers to the presence of conspicuous bracteoles at the base of the female bracts, a feature that is relatively common in *Acalypha* species from Madagascar but infrequent in continental African species.

3.1.3. Acalypha linearis

Acalypha linearis I.Montero, Cardiel & P.Muñoz, *sp. nov*. Type: Angola, Bié Province, Cuemba, 17 km from Munhango, [12º08’43’’S 18º05’11’’E], 19 Sep 1965. *R. Mendes dos Santos 1881* (LISC[LISC052457], holo.). Fig. 5.

Diagnosis. Acalypha linearis is primarily characterised by its numerous unbranched deeply striate stems arising from a woody rootstock; sessile, linear to aciculiform leaves with entirely or partially revolute margins; and terminal female inflorescences up to 3 cm long,

with bracts densely pubescent with simple trichomes, and margins regularly dentate with 7−9 teeth.

Description. Perennial herb up to 35 cm tall, with many unbranched stems from a woody rootstock, monoecious or dioecious. Stems deeply striate, subglabrous, with some short, simple trichomes towards the apex. Stipules conspicuous, up to 2.5 mm long, narrowly triangular to linear-lanceolate, arched, pubescent with simple, short trichomes c. 0.6 mm long. Leaves sessile. Leaf blades (2.5−)3−8.5(−9) × 0.1−0.2(−0.3) cm, linear to aciculiform, chartaceous; apex acute, slightly callose; margins totally or partially revolute, mostly entire, denticulate towards the apex; upper and lower surface glabrous; venation pinnate, secondary veins inconspicuous, c. 10 per side. Inflorescences unisexual, spiciform, usually in different stems, male axillary, female terminal. Male inflorescences up to 3.5 cm long; peduncle up to 1.3 cm long, glabrous; flowers glomerate; bracts up to 1.2 mm long, linear to subspathulate, pubescent, with short, simple trichomes c. 0.5 mm long. Female inflorescences up to 3 cm long; peduncle up to 1.2 cm long, pubescent with some short, simple, slender trichomes c. 1 mm long, glabrescent; bracts 7−8, sessile, enlarging in fruit up to 5.5 × 7 mm, minutely papillose, densely pubescent with short, simple, trichomes c. 0.5 mm long; margin regularly dentate, teeth 7−9, triangular, acute, ½–⅓ of the total length; bracteoles absent. Male flowers with pedicel up to 1 mm long, sparsely hairy with short, simple trichomes c. 0.7 mm long; buds c. 0.8 mm diameter, sparsely hairy with simple, short, slender trichomes c. 0.7 mm long, mainly distally. Female flowers 1 per bract, sessile or subsessile; sepals 3, up to 1.2 mm long, distinct, oblong-lanceolate, ciliate with short, simple trichomes c. 0. 5 mm long; ovary c. 1 mm diameter, 3-lobed, densely hispid with simple, short trichomes; styles 3, up to 11 mm long, slightly connate at base, each with 8–15 short, simple branches, sparsely hairy with some, short, simple trichomes at rachis. Capsules up to 6 mm diameter, surface smooth,

densely pubescent with short, simple trichomes c. 0.7 mm long, mainly towards the apex. Seeds c. 3 × 2.1 mm, pyriform, minutely foveolate.

Additional specimens. Angola. Bié Province: General-Machado [Camacupa], 1 km from Savinguila do Cuemba, 1100 m, [12º01’03’’S 17º28’24.9’’E], 4 Oct 1965. *B. Teixeira et al. 8971* (LISC052360, LISC052530); General-Machado, [Camacupa], Munhango, 1100 m, [12º09’28’’S 18º33’20’’E], 13 Sep 1965. *B. Teixeira et al. 8854* (LISC052361, LISC052530); Cuemba, riverbank of Cuiba river, 36 km from Decumba, [12º08’43’’S 18º05’11’’E], 27 Aug 1965. *R. Mendes dos Santos 1732* (LISC052475); Cuemba, [12º08’43’’S 18º05’11’’E], 13 Sep 1965. *R. Monteiro e Murta 1713* (LISC052522); Cuiba, in the Kalahari sands, [12º07’01’’S 17º57’32’’E], 27 Aug 1965. *R. Monteiro e Murta 1564* (LISC052525); Camacupa, Cuemba, Cuiva, 1200 m, [12º07’01’’S 17º57’32’’E], 27 Aug 1965. *B. Teixeira & R. Monteiro e Murta 8732* (LISC052529).

Distribution and habitat. Acalypha linearis appears to be endemic to Angola and is known only from Bié Province, where it occurs at an altitude of approximately 1,100 m (Fig. 3). It inhabits the Miombo woodlands ecoregion (Huntley, 2019), particularly in ecotonal areas characterised by shrub formations and sandy soils.

Preliminary conservation status. *Acalypha linearis* is currently known from seven collections, all made in Bié province, Angola, between August and September 1965. These collections originate from closely located sites, representing a single threat-based location. With no recent records available, the current population status remains unknown. We estimate the species’ EOO at approximately 344 km², and its AOO at 16 km².
All collections were made outside Angola’s protected areas network. *Acalypha linearis* occurs in woodlands and open forests, ecosystems that are increasingly threatened by agricultural expansion (Barstow, 2023; Mendelsohn and Sohngen, 2019). The ongoing

conversion of these habitats may lead to a decline in habitat quality, potentially reducing the species' viability. Based on its restricted distribution, lack of recent records, and increasing habitat threats, we preliminarily assess *A. linearis* as Endangered (EN) under IUCN criteria B1ab(iii)+2ab(iii).

Etymology. The epithet refers to the characteristically sessile, linear leaves.

4. DISCUSSION

The three new species described here belong to *Acalypha* subgenus *Acalypha*. Species in this subgenus can be easily recognised within *Acalypha* by their sessile or subsessile female flowers, calyx with 3 sepals, and generally accrescent, foliaceous bracts in fruit (Levin et al., 2022).

Acalypha brevipetiolata is part of a strongly supported but geographically widespread African clade that currently includes *A. cupricola, A. manniana*, *A. polymorpha*, and *A. welwitschiana* (Fig. 2; Table 1). Additional species may belong to this clade as more sequencing is conducted. Of the currently included species, only *A. polymorpha* and *A. welwitschiana* have been recorded in Tanzania. *Acalypha cupricola* is endemic to the Democratic Republic of the Congo, while *A. manniana* is more widespread, occurring in neighbouring countries such as Uganda, Rwanda, and Burundi, though it has not been recorded in Tanzania. The phylogenetic affinity of *A. brevipetiolata* with these species suggests a broader African lineage that remains understudied.

Morphologically, *Acalypha brevipetiolata* bears a superficial resemblance to *A. linearifolia* Leandri, a species endemic to Madagascar's littoral forests and thickets (Montero-Muñoz et al., 2023). However, beyond their occurrence in different habitats, they also differ in key diagnostic features of their habit (shrubby in *A. linearifolia* vs. herbaceous in *A. brevipetiolata*), female bracts (with stellate trichomes and entire to denticulate margins in *A.*

linearifolia vs. with simple trichomes and deeply dentate margins in *A. brevipetiolata*), and capsules (papillose with stellate trichomes in *A. linearifolia* vs. smooth with simple trichomes in *A. brevipetiolata*). The absence of molecular data for *A. linearifolia* prevents direct phylogenetic comparison.

Acalypha linearis, the new species from Angola, resembles *A. brevipetiolata*, particularly in habit and leaf shape, but differs in other key characters already mentioned in the descriptions. Both species appear to be geographically restricted to opposite sides of the continent, with *A. linearis* likely occurring at slightly lower elevations. Both species are associated with fire-prone ecosystems characterised by marked seasonality and sandy soils (Huntley, 2019) and —along with several other African *Acalypha* representatives— possess a woody rootstock, an adaptation commonly linked to fire resilience (Sebata, 2017). Given that this trait is also present in other members of the *A. brevipetiolata* clade, it would not be surprising if molecular analyses reveal a close relationship between *A. linearis* and its sequenced counterpart. These findings suggest that certain *Acalypha* lineages have undergone ecological adaptations to seasonally dry and fire-influenced habitats across Africa.

Finally, ***Acalypha bracteolata***, the other Tanzanian species described here, is morphologically similar to *A. ornata* Hochst. ex A.Rich., a widespread African species, but differs in key traits, including their habit (herbaceous in *A. bracteolata* vs. shrubby in *A. ornata*), female inflorescences (up to 9.5 cm long in *A. bracteolata* vs. up to 15 cm long in *A. ornata*) and developed female bracts (up to 6.2 × 4 mm with conspicuous bracteoles in *A. bracteolata* vs. up to 12 × 11 mm and without bracteoles in *A. ornata*). However, due to the limited knowledge of the genus in Africa, its phylogenetic placement remains uncertain without molecular data.

This highlights a broader challenge: our understanding of African *Acalypha* is constrained by the overall lack of both morphological and molecular studies. As a result, it is often difficult to determine whether observed similarities reflect shared ancestry or convergent evolution in similar ecological settings.

Of the three species, we obtained high-quality DNA and successfully sequenced *Acalypha brevipetiolata*, allowing its placement in a phylogenetic framework. The evolutionary relationships of *A. bracteolata* and *A. linearis* remain uncertain due to the lack of molecular data, highlighting the need for further sequencing efforts. Future phylogenetic studies incorporating additional African taxa will be essential to resolve their placement within the genus and clarify species boundaries.

4.1. Conservation

The two new Tanzanian species—*A. brevipetiolata* and *A. bracteolata*—are currently known from only a single collection each, and their population status remains unknown. This represents a major limitation in assessing their conservation status. Similarly, *A. linearis* is known from seven collections but they were all made in 1965 in the same Angolan region, thus representing a single threat-based locality. To the best of our knowledge, this species has not been recorded in over sixty years, leaving its current population status uncertain. Given the rarity of these species and their restricted known distributions, urgent field surveys are needed to locate additional populations, assess potential threats, and evaluate their conservation status.

4.2. Taxonomic implications

As noted by Cardiel et al. (2025), species descriptions for mainland Africa have been rare in recent decades, with only two new *Acalypha* species published in the last 30 years (Levin et

al., 2007) —compared to 30 from the Americas, 7 from Asia, and 17 from Madagascar and surrounding islands over the same period. The gap is even more striking for Tanzania and Angola: *A. gillmanii* Radcl.-Sm., the last species described from Tanzanian material, was published in 1975, while no new species have been described from Angolan specimens since Pax and Hoffman's *Das Pflanzenreich* a century ago (Pax and Hoffmann, 1924). In this context, our description of three new species represents the first formal addition to these countries' *Acalypha* flora in decades. This underscores the need for continued botanical exploration and taxonomic revision in the region.

Ongoing taxonomic work, including herbarium research, molecular studies and field surveys, is critical to delimiting species boundaries and elucidating evolutionary relationships within *Acalypha*. In-person visits to herbaria —both within Tanzania and Angola and in former colonial institutions where their historical collections are housed— along with targeted specimen loans remain essential to our work. These efforts will contribute to a more comprehensive understanding of *Acalypha* diversity and biogeography in Africa, a region where we anticipate many *Acalypha* species have yet to be described.

FUNDING

The authors are grateful for the financial support provided by the research project PID2022-139634NA-I00M funded by MCIN/AEI/10.13039/501100011033/FEDER, UE. PMR was supported by a Ramón y Cajal research contract, grant ref. RYC2021-032489-I, and IMM by a Juan de la Cierva – Formación-2021, grant ref. FJC2021-046607-I, funded by MCIN/AEI/ 10.13039/501100011033 and the European Union NextGenerationEU/PRTR during the work on this project.

CRediT AUTHORSHIP CONTRIBUTION STATEMENT

Iris Montero-Muñoz: Conceptualization, Data curation, Investigation, Validation, Visualization, Writing – original draft, Writing – review & editing. **José María Cardiel**: Conceptualization, Data curation, Investigation, Validation, Visualization, Writing – original draft, Writing – review & editing. **Lucía Villaescusa-González**: Formal analysis. **Pablo Muñoz-Rodríguez**: Conceptualization, Data curation, Investigation, Validation, Visualization, Writing – original draft, Writing – review & editing, Project administration, Funding acquisition.

DECLARATION OF COMPETING INTEREST

All authors declared that they have no competing interest.

ACKNOWLEDGEMENTS

The authors would like to acknowledge the herbarium curators at BR, K, LISC, LISU, MA, MO, P, and WAG for access to their collections.

REFERENCES

Bachman, S., Moat, J., Hill, A., de la Torre, J., Scott, B., 2011. Supporting Red List threat assessments with GeoCAT: geospatial conservation assessment tool. ZooKeys 150, 117–126. https://doi.org/10.3897/zookeys.150.2109

Barstow, M., 2023. Monotes paivae: Barstow, M.: The IUCN Red List of Threatened Species 2023: e.T137436555A222511392. https://doi.org/10.2305/IUCN.UK.2023-1.RLTS.T137436555A222511392.en

Burgess, N.D., Butynski, T.M., Cordeiro, N.J., Doggart, N.H., Fjeldså, J., Howell, K.M., Kilahama, F.B., Loader, S.P., Lovett, J.C., Mbilinyi, B., Menegon, M., Moyer, D.C., Nashanda, E., Perkin, A., Rovero, F., Stanley, W.T., Stuart, S.N., 2007. The

biological importance of the Eastern Arc Mountains of Tanzania and Kenya. Biol. Cons. 134, 209–231. https://doi.org/10.1016/j.biocon.2006.08.015

Capella-Gutierrez, S., Silla-Martinez, J.M., Gabaldon, T., 2009. trimAl: a tool for automated alignment trimming in large-scale phylogenetic analyses. Bioinformatics 25, 1972–1973. https://doi.org/10.1093/bioinformatics/btp348

Cardiel, J.M., 1995. *Acalypha* (Euphorbiaceae), Flora de Colombia. Universidad Nacional de Colombia y Real Jardín Botánico de Madrid – CSIC. https://repositorio.unal.edu.co/handle/unal/80876

Cardiel, J.M., Montero-Muñoz, I., Gamarra, R., Ortúñez, E., Muñoz-Rodríguez, P., 2025. A nomenclatural review of *Acalypha* L. (Euphorbiaceae) of mainland Africa. Taxon in press.

Cardiel, J.M., Muñoz-Rodríguez, P., González-Berdasco, Á., Montero-Muñoz, I., 2023. Catalogue and red list of *Acalypha* L. (Euphorbiaceae) from South America. Eur. J. Taxon. 886, 1–92. https://doi.org/10.5852/ejt.2023.886.2201

Cheng, T., Xu, C., Lei, L., Li, C., Zhang, Y., Zhou, S., 2016. Barcoding the kingdom Plantae: new PCR primers for *ITS* regions of plants with improved universality and specificity. Mol. Ecol. Res. 16, 138–149. https://doi.org/10.1111/1755-0998.12438

Crawford, F.M., Darbyshire, I., Vollesen, K., 2016. Tephrosia uzondoensis (Leguminosae: Papilionoideae): a new species from the Uzondo Plateau of Tanzania. Kew Bull. 71, 12. https://doi.org/10.1007/s12225-016-9621-1

Doggart, N., Meshack, C.K., 2023. Situation analysis of the Udzungwa Mountains landscape. Tanzania Forest Conservation Group.

Doyle, J.J., Doyle, J.L., 1990. Isolation of plant DNA from fresh tissue. Focus 12, 13–15.

Figueiredo, E., Smith, G.F., 2008. Plants of Angola, Strelitzia. South African National Biodiversity Institute (SANBI), Pretoria.

Huntley, B.J., 2019. Biodiversity of Angola. Science and conservation: a modern synthesis, 1st ed. Springer Nature Publishing AG, Cham, Switzerland.

IUCN, 2022. Guidelines for using the IUCN Red List categories and criteria. Version 15. Standards and Petitions Subcommittee, IUCN, Gland, Switzerland and Cambridge, UK.

Kalyaanamoorthy, S., Minh, B.Q., Wong, T.K.F., von Haeseler, A., Jermiin, L.S., 2017. ModelFinder: fast model selection for accurate phylogenetic estimates. Nat. Methods 14, 587–589. https://doi.org/10/gfzprj

Katoh, K., Standley, D.M., 2013. MAFFT multiple sequence alignment software version 7: improvements in performance and usability. Mol. Biol. Evol. 30, 772–780. https://doi.org/10.1093/molbev/mst010

Levin, G.A., Cardinal-McTeague, W.M., Steinmann, V.W., Sagun, V.G., 2022. Phylogeny, classification, and character evolution of *Acalypha* (Euphorbiaceae: Acalyphoideae). Syst. Bot. 47, 477–497. https://doi.org/10.1600/036364422X16512572275034

Levin, G.A., Morton, J.K., Robbrecht, E., 2007. Two new species of *Acalypha* (Euphorbiaceae) from Tropical Africa, and a review of some robyns names for cupricolous plants from the Democratic Republic of the Congo. Syst. Bot. 32, 576–582. https://doi.org/10.1600/036364407782250599

Mahunnah, R.L.A., 1995. Taxonomy, phytogeography and ethnobotany of the genus *Acalypha* L. (Euphorbiaceae) in Tanzania (Doctoral Thesis). University of Dar es Salaam, Dar es Salaam.

Mendelsohn, R., Sohngen, B., 2019. The net carbon emissions from historic land use and land use change. J. Forest Econ. 34, 263–283. https://doi.org/10.1561/112.00000505

Montero-Muñoz, I., Levin, G.A., Cardiel, J.M., 2023. Monograph of *Acalypha* L. (Euphorbiaceae) of the Western Indian Ocean Region, with the description of a new species from Mayotte. Adansonia 45. https://doi.org/10.5252/adansonia2023v45a26

Nguyen, L.-T., Schmidt, H.A., von Haeseler, A., Minh, B.Q., 2015. IQ-TREE: a fast and effective stochastic algorithm for estimating Maximum-Likelihood phylogenies. Mol. Biol. Evol. 32, 268–274. https://doi.org/10.1093/molbev/msu300

Pax, F., Hoffmann, K., 1924. Euphorbiaceae-Crotonoideae-Acalypheae-Acalyphinae, in: Engler, H.G.A. (Ed.), Das Pflanzenreich. Verlag von Wilhelm Engelmann, Leipzig, Germany, pp. 1–231.

Radcliffe-Smith, A., 1987. Euphorbiaceae, in: Polhill, R.M. (Ed.), Flora of Tropical East Africa. A.A.Balkema, Rotterdan & Boston, p. 407.

Sagun, V.G., Levin, G.A., van Welzen, P.C., 2010. Revision and phylogeny of *Acalypha* (Euphorbiaceae) in malesia. Blumea 55, 21–60. https://doi.org/10.3767/000651910X499141

Sebata, A., 2017. Ecology of woody plants in African savanna ecosystems, in: Yousaf, Z. (Ed.), Plant Ecology - Traditional Approaches to Recent Trends. IntechOpen, pp. 25–42. https://doi.org/10.5772/intechopen.69865

Silva, O.L.M., Riina, R., Cordeiro, I., 2020. Phylogeny and biogeography of *Astraea* with new insights into the evolutionary history of Crotoneae (Euphorbiaceae). Mol. Phyl. Evol. 145, 106738. https://doi.org/10.1016/j.ympev.2020.106738

Thiers, B., 2025. Index Herbariorum: a global directory of public herbaria and associated staff. New York Botanical Garden’s Virtual Herbarium.

Wells, T., Carruthers, T., Muñoz-Rodríguez, P., Sumadijaya, A., Wood, J.R.I., Scotland, R.W., 2021. Species as a heuristic: reconciling theory and practice. Syst. Biol. 71, 1233–1243. https://doi.org/10.1093/sysbio/syab087

Figure 1. *Acalypha brevipetiolata*. A. Habit of female branch. B. Habit of male branch. C. Detail of lower leaf surface. D. Detail of node, stipules, and female flower. E. Mature female bract. F. Ovary and styles. G. Calyx of the female flower. H. Capsule. I. Seed. Based on *S. Bidgood, G. Leliyo & K. Vollesen 8090*. Illustration by Laura González Hernández.

Figure 2. *nrITS* phylogeny of *Ipomoea* showing the position of *Acalypha brevipetiolata* within a clade alongside four other species. The remaining clades, named following Levin et al. (2022), have been collapsed to enhance readability. The full phylogeny is available in Supplementary fig. 1.

Figure 3. Map of Angola (A) and Tanzania (B) showing the collection sites of the new species. Blue, *Acalypha linearis* (seven collections); red, *A. brevipetiolata* (one collection); black, *A. bracteolata* (one collection).

Figure 4. *Acalypha bracteolata*. A. Habit. B. Detail of node, stipules, petiole base and male inflorescence base. C. Detail of base of lower leaf surface. D. Detail of leaf margin. E. Mature female bract. F. Mature male bract G. Ovary and styles. H. Calyx of the female flower. Based on *G. Massawe 282*. Illustration by Laura González Hernández.

Figure 5. *Acalypha linearis*. A. Habit. B. Detail of a branch with male inflorescences. C. Detail of node, stipules and leaf base. D. Detail of apex of lower leaf surface. E. Mature female bract. F. Ovary and styles. G. Calyx of the female flower. H. Capsule. I. Seed. Based on *R. Mendes dos Santos 1881*. Illustration by Laura González Hernández.

Table 1. Key morphological characters distinguishing species within the same clade as *Acalypha brevipetiolata*.

figure 1

Click here to access/download;Figure;figure1.png

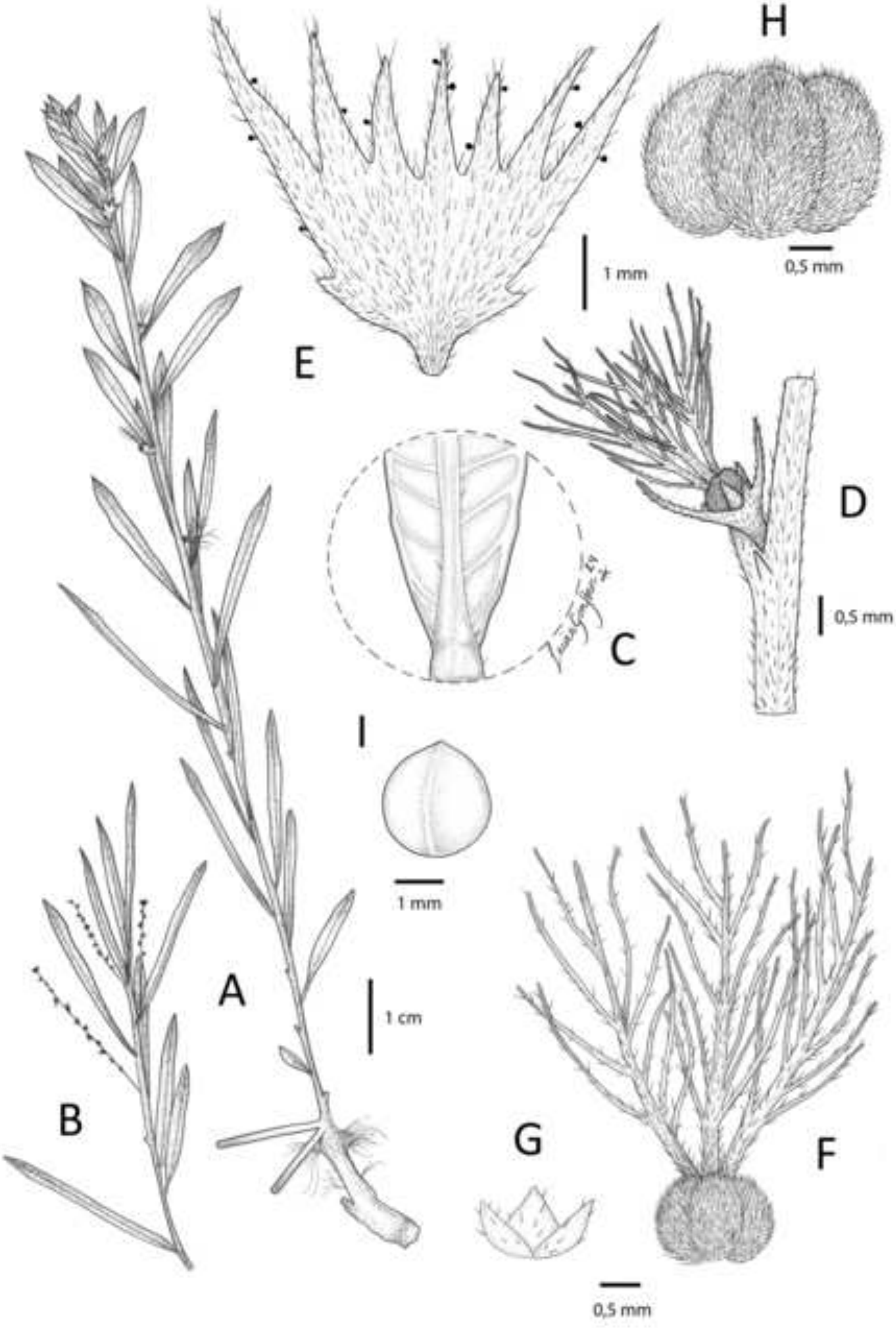

figure 2

Click here to access/download;Figure;figure2.jpg

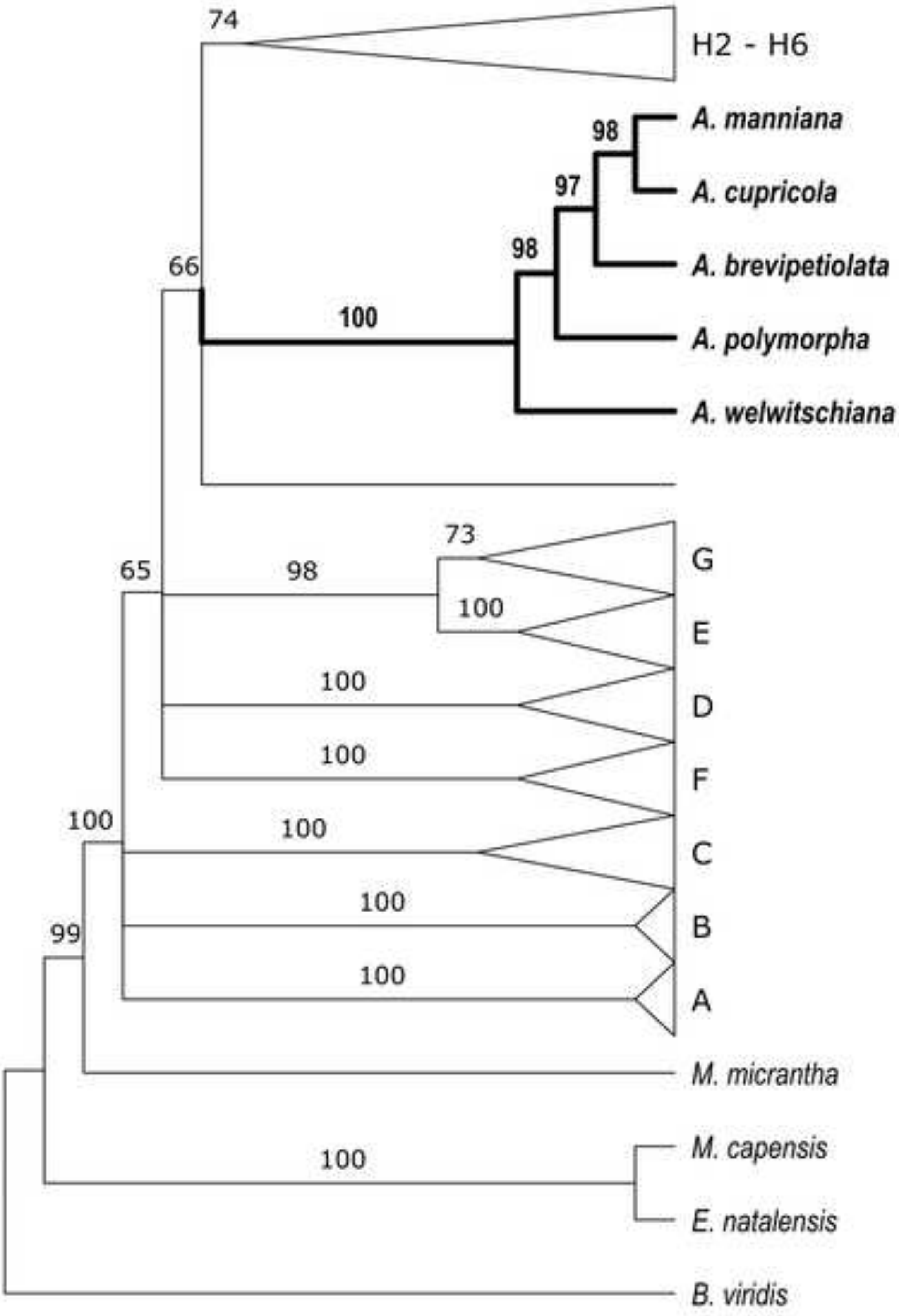

figure 3
Click here to access/download;Figure;figure3.jpg

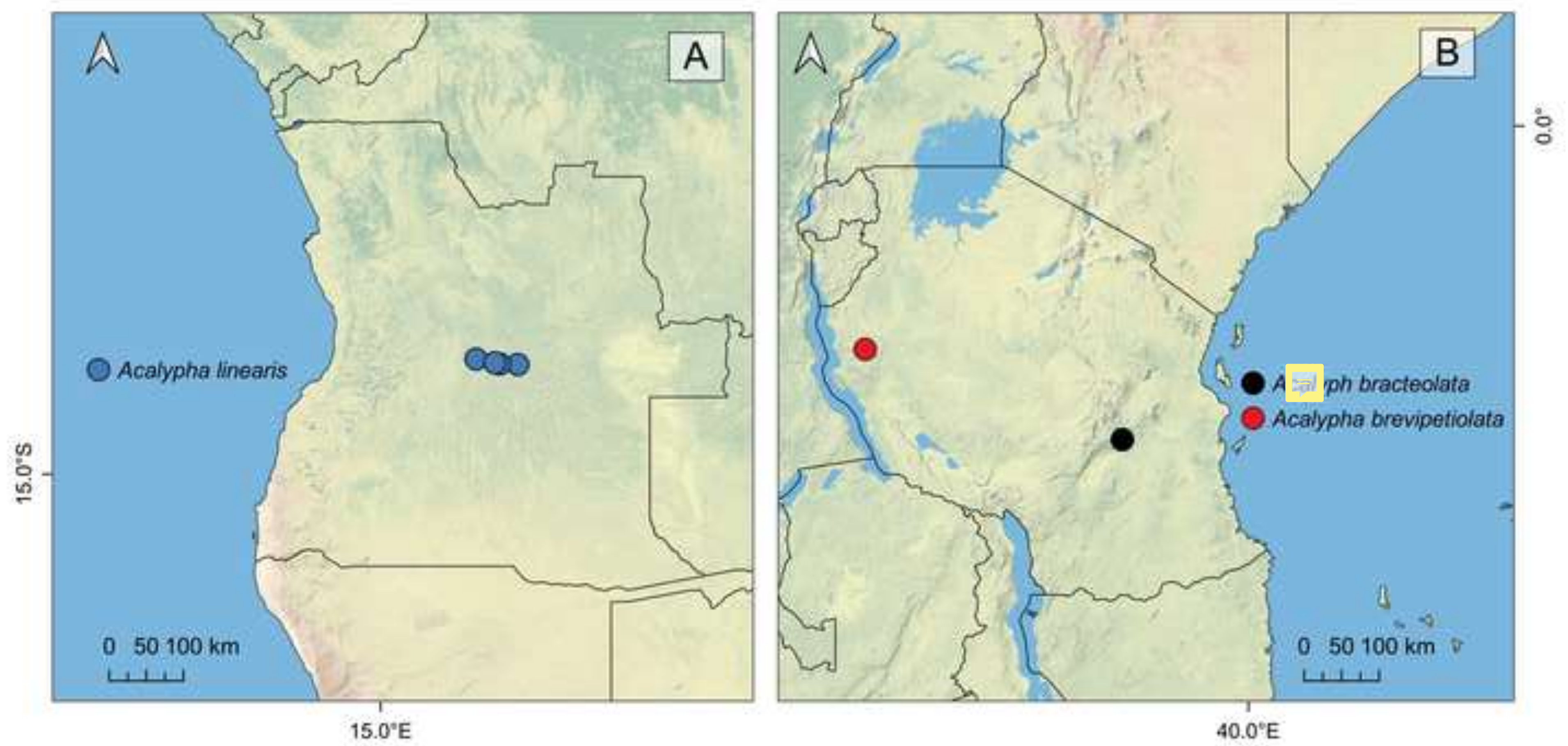

Click here to access/download;Figure;figure4.png

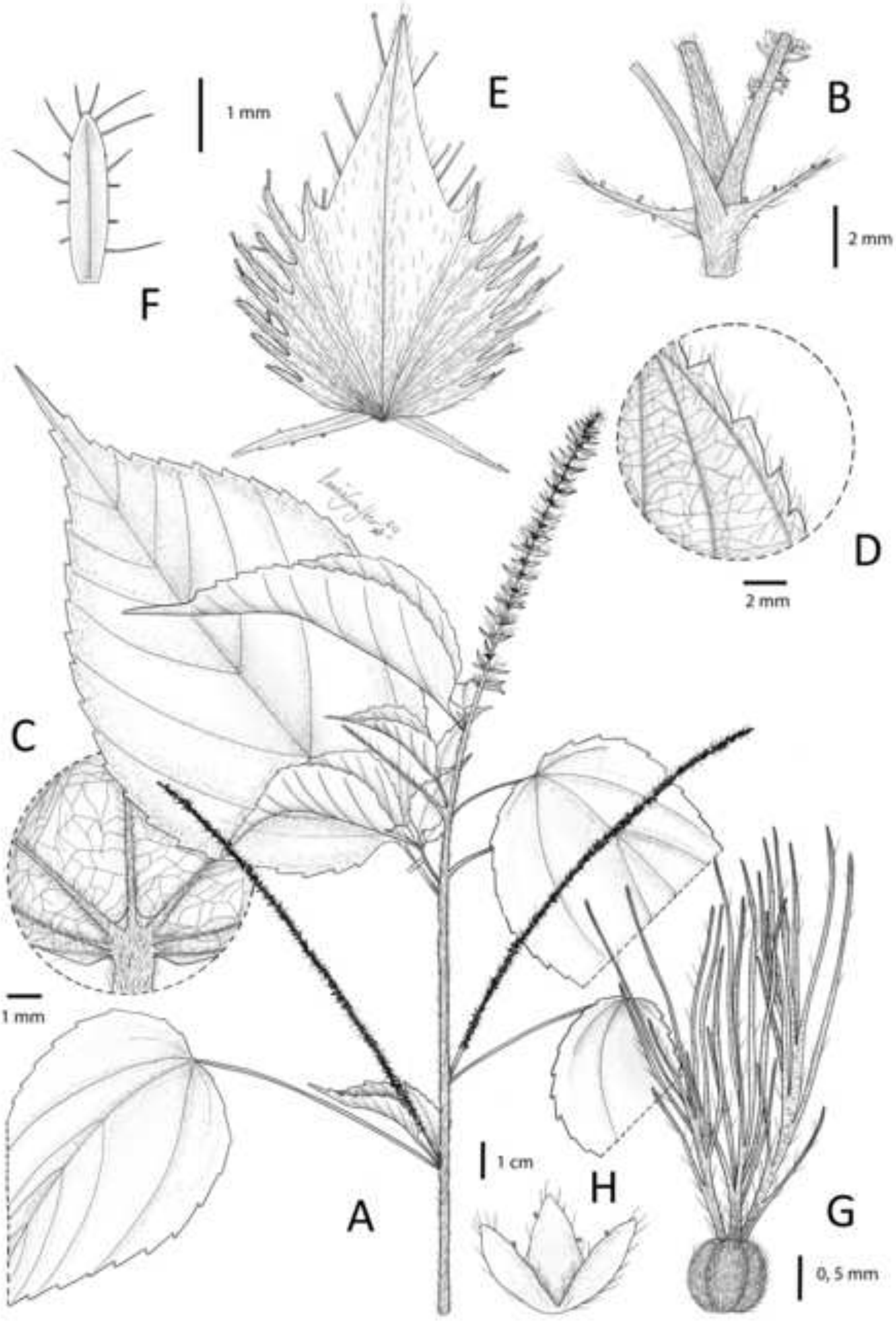

figure 5

Click here to access/download;Figure;figure5.png

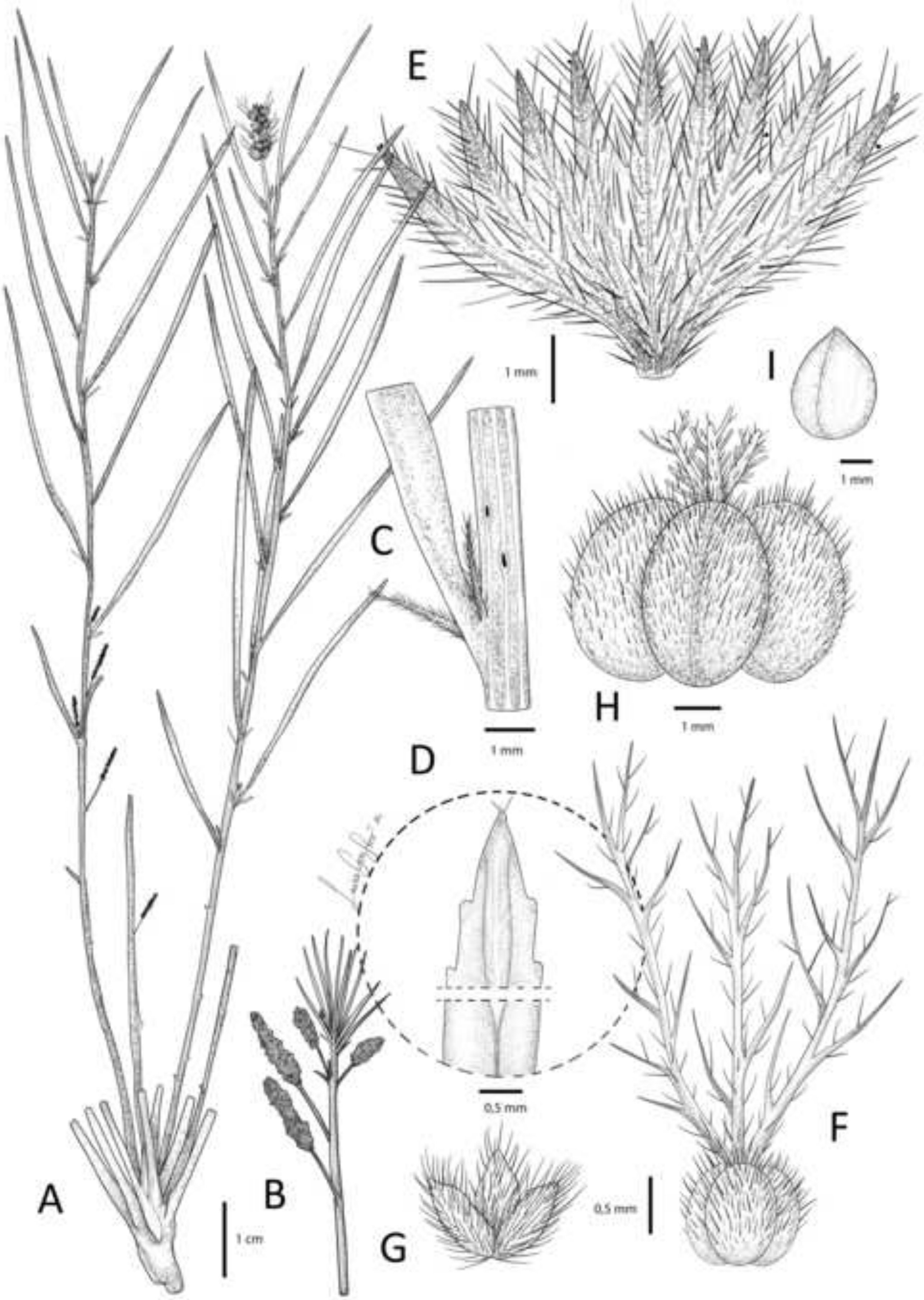

supplementary figure 1

Click here to access/download
Supporting Information
suppl_figure1.svg

supplementary file 1

Click here to access/download
Supporting Information
suppl_file_1.docx

Click here to access/download
Supporting Information - Compressed/ZIP File Archive
phylogenetic_analysis.zip
