# Supplementary material for: Three new species of Acalypha L. (Euphorbiaceae, Acalyphoideae) from Tanzania and Angola and their conservation status
Source: PLoS One. 2025 Oct 29;20(10):e0332588. doi: 10.1371/journal.pone.0332588 (PMC12571263; doi:10.1371/journal.pone.0332588)
Supplement: Attachment — Submitted filename: rebuttal_letter.pdf [file pone.0332588.s006.pdf]

UNIVERSIDAD
COMPLUTENSE
MADRID

Pablo Muñoz-Rodríguez
Faculty of Biological Sciences
Department of Biodiversity, Ecology and Evolution

21st July 2025

Dear Editor,

Thank you for considering our manuscript for publication in PLOS One. We submit a revised version of the manuscript addressing all reviewer's suggestions, and provide comments to two reviewer's comments and to your editorial comments below.

Reviewer's comments:

- "Why was dimethyl sulfoxide (DMSO) not included in the amplification to prevent the formation of secondary structures during PCR?"

DMSO has not shown significant improvement in amplification success in our extensive work on *Acalypha*. The primers, annealing temperatures, and template concentrations are optimised to minimise secondary structures, as demonstrated in all our previous studies.

- Addition of authorities in the names in the map in Figure 3.

We have chosen not to include authorities in Figure 3 to preserve clarity and readability, due to the limited space and visual density of the map. Full scientific names with authorities are provided in the taxonomic treatment and in the captions of the specimen illustrations and the map, ensuring proper attribution without compromising the figure's visual effectiveness.

Editorial comments:

- New "Nomenclature" sub-section in the Methods section.

As requested, we have included a new sub-section with the text indicated.

- GUID identifiers

We have included GUID chains in all three names, with *XXXX* as placeholders pending assignment of identifiers by IPNI

- Funding statement.

All relevant funding has been declared. We have updated the funding statement to include the statement indicated at the end. The new funding statement reads as follows:

C/ José Antonio Novais, 12. 28040 Madrid, SPAIN.

UNIVERSIDAD
COMPLUTENSE
MADRID

Pablo Muñoz-Rodríguez
Faculty of Biological Sciences
Department of Biodiversity, Ecology and Evolution

The authors are grateful for the financial support provided by the research project PID2022-139634NA-I00M funded by MCIN/AEI/10.13039/501100011033/FEDER, UE. PMR was supported by a Ramón y Cajal research contract, grant ref. RYC2021-032489-I, and IMM by a Juan de la Cierva – Formación-2021, grant ref. FJC2021-046607-I, funded by MCIN/AEI/ 10.13039/501100011033 and the European Union NextGenerationEU/PRTR during the work on this project. There was no additional external funding received for this study.

- Data availability.

We would like to clarify that we explicitly stated in both the online submission form and the manuscript that the DNA sequence data have already been submitted to GenBank, with accession numbers provided in the paper, and are scheduled for public release upon publication. All other data, including analyses and Supporting Information files, were submitted with the manuscript and are fully available within the submission. This ensures full compliance with PLOS One data availability policy, as the complete dataset will be openly accessible upon publication.

- Acknowledgement section.

Unless we are mistaken, our Acknowledgments section contains no mention of funding. The only statement included is:

"The authors would like to acknowledge the herbarium curators at BR, K, LISC, LISU, MA, MO, P, and WAG for access to their collections."

All funding information was provided exclusively in the Funding Statement, in accordance with the journal's guidelines.

- Table 1.

As requested, we have included Table 1 at the end of the main manuscript file.

- Supporting information.

We have included captions for Supplementary Figure 1, Supplementary Table 1 and Supplementary File 1 at the end of the manuscript. In the text, we named them 'S1

UNIVERSIDAD
COMPLUTENSE
MADRID

Pablo Muñoz-Rodríguez
Faculty of Biological Sciences
Department of Biodiversity, Ecology and Evolution

Figure', 'S1 Table' and 'S1 File' respectively. Please confirm if this terminology is acceptable or if an alternative format is preferred.

- Map in Figure 3.

We have replaced the basemap in figure 3 for a CC-BY CGRI layer. A reference has been added to the Methodology section and the Reference list (*Jarvis, A., Reuter, H.I., Nelson, A., Guevara, E., 2008, Hole-filled SRTM for the globe Version 4, available from the CGIAR-CSI SRTM 90m Database (http://srtm.csi.cgiar.org)*).

Thank you very much for considering our submission.

Best regards,

Pablo Muñoz-Rodríguez, DPhil, FLS

(on behalf of all authors)

C/ José Antonio Novais, 12. 28040 Madrid, SPAIN.
